# Economic and Efficient phosphonic functional groups mesoporous silica for uranium selective adsorption from aqueous solutions

**DOI:** 10.1038/s41598-019-46090-2

**Published:** 2019-07-04

**Authors:** H. Sarafraz, Gh. Alahyarizadeh, A. Minuchehr, H. Modaberi, A. Naserbegi

**Affiliations:** 1 Engineering Department, Shahid Beheshti University, G.C., P.O. Box 1983969411, Tehran, Iran; 2 Academic Center for Education, Culture and Research (ACECR), Environmental Research Institute, Siadati Street, Mellat Avenue, P.O. Box 3114-41635, Rasht, Iran

**Keywords:** Natural hazards, Characterization and analytical techniques

## Abstract

A novel mesoporous silica with enhanced phosphonic functional groups (PFGs) and without any silane agents was provided by Sol-Gel technique for selective adsorption of U(VI) from aqueous solutions (AqS). The absorbent was synthesized based on the achieving the four best performance characteristics including availability, economically, adsorption efficiency, and selectivity which are undoubtedly defined the usefulness of the adsorbents. The sorption results exhibited the highest uranium adsorption capacity, qe, of 820.7 mg/g at pH ≈ 8 which indicated the adsorbent is the best alternative for uranium adsorption from the nearly neutral solutions such as seawater. The recovery percentages by the adsorbent in the aqueous environments involving other elements such as Mg, Cd, Hg, As, Ca, Na, Ni and the salty environment with high concentration of Cl^−^ ions are indicated that the adsorbent presents the acceptable selectivity for uranium adsorption from the AqS such as industrial wastewater. Several activities and factors including removing the silane agents, using sodium metasilicate as an available and low-cost source of silica, and using the Sol-gel method as an unexpansive synthesis technique caused to reduce the synthesis costs from 222.787 EUR/kg for the template method to 60.078 EUR/kg for Sol-gel method which confirm the synthesis of a cost-effective adsorbent.

## Introduction

Adsorption is one of the most successful technique, among the biological and physio-chemical techniques which widely used for removal heavy metals like uranium from AqS. In addition to low capital investment and operating cost, this method presents the lowest generation of solid waste^1,2^. Several different kinds of adsorbents such as clays, polymers, resins, activated carbons, activated alumina, magnetite nanoparticles and nanocomposite, and zeolites have been synthesized, improved, and examined for U adsorption from AqS. Several problems have to consider in the case of adsorbent selection like the mechanical and thermal stability and the type of chemical union with the metals^3,4^.

Ordered mesoporous silica’s (MSs) are one of the most promising materials which satisfy above-mentioned criteria and widely applied for ion separation purposes in the environmental treatment, chemical and biological processes, metallurgical purposes, and nuclear engineering. Up to now, numerous MSs have been introduced as the adsorbents chiefly to remove uranium from AqS and wastewaters. Uranium is known as the most hazardous material among heavy metal cations. One of the main parameters which affect MSs efficiencies is functional groups. Among several functional groups, phosphonic acid derivatives are the efficient functional groups in porous silica’s which enhance considerably qe and selectively extract uranium from the AqS. These properties lead to introduce phosphonic acid functional groups MSs as the best potential in sorption technologies. Investigation of MS functional group present different properties of sorbates such as adsorption capacity, active sites, accessibility^5–9^.

Yu-Long Wang *et al*. investigated the sorption behaviors of the different functionalized SBA-15 on uranium removal from aqueous solution. They synthesized functional SBA-15 by post-grafting method by using 2-diethylethylphosphonate triethoxysilane (DPTS) as the silane coupling agents and ethylphosphonic acid (PA). Their results indicated that maximum uranium qe was obtained of 0.914 mmol/g for SBA-15-PA compared with 0.339 mmol/g for SBA-15^10^. Xiao Liang Wang *et al*. were also used post-synthesis grafting method to prepare the phosphonate-amino bi-functionalized mesoporous silica SBA-15 as a uranium sorbent in batch process. The silane coupling agents which were used for functionalizing of SBA-15 were Aminopropyltriethoxysilane (APS) and DPTS. They proclaimed uranium qe of 244 mg/g at pH 5.5 under room temperature^11^. Xue Guo *et al*. synthesized phosphoryl functionalized MS (TBP-SBA-15) by modifying MS with tributyl phosphate (TBP) and NH_2_-SBA-15 with γ-aminopropyl triethoxy silane. Their results revealed that the uranium qe was enhanced from the 140 mg/g to 200 mg/g for TBP-SBA-15 and 190 mg/g for NH_2_-SBA-15^12^. Chamila Gunathilake *et al*. used the hydroxyphos-Phatoethyl pendant groups as the functional groups for modifying mesoporous silica materials. They synthesized hydroxyphos-Phatoethyl mesoporous silica (POH-MS) by a two-step process including copolymer template synthesis and surface modification. They used synthesized POH-MS for removal lead from aqueous solution and reached the highest qe of 272 mg/g^13^. Magnetite phosphonic acid functionalized silica microspheres were synthesized by Limin Zhou *et al*. to remove uranium from AqS. They used DPTS as the coupling agent by which obtained the maximum uranium qe of 76.9 mg/g^14^. Different functional groups MS’s were prepared by A. Dudarko *et al*. They synthesized the various adsorbents based on altering the sodium metasilicate and DPTS molar ratios. The highest uranium qe reported by this research group was of 54.5 mg/g for 10:2 molar ratio of the sodium metasilicate and DPTS^15^. In our previous work, DPTS and phosphoric acid were used to enhance the PFGs of MSs which cause to considerable increase of uranium capacity to 207 mg/g^16^.

Although, several MSs with different functional groups were synthesized by different research teams, researchers try to find and improve MSs with better performance characteristics. Certainly, availability, economically, adsorption efficiency, and selectivity are four main performance characteristics which determine the suitability of the adsorbents.

This study presents an economical MS with enhanced PFGs synthesized by Sol-Gel process to selectively adsorb uranium [U(VI)] from AqS. The main approach in this work was based on achieving the best performance characteristics, i.e. use of accessible and cost-effectiveness materials in the synthesis process which causes to prepare economic adsorbents, achieving higher adsorption efficiency by enhancing functional groups, which lead to selective adsorption of uranium. As well as, several key parameters which have an impact on the qe, including pH, volume and initial U(VI) concentration of solution, added adsorbent concentration and contact time were studied. Regarding the advantage of phosphoric acid as an unexpansive source of the PFG, several achievements were anticipated compared to earlier reported studies such as (1) enhanced PFGs which lead to heightened uranium adsorption, (2) Removal of silane agents, (3) Use of Sodium metasilicate as a low-cost source of silica, (4) Use of Sol-gel method as a cheap technique (it doesn’t need template polymer and synthesis time is short), (5) selective adsorption of uranium.

## Experimental Section

### Chemicals

Different materials, solutions and standards were used in synthesis procedures and adsorption tests which are Sodium metasilicate, Na_2_SiO_3_·9H_2_O (Sigma, USA), TEOS (98%; Aldrich, USA), orthophosphoric acid (85%; Merck, Germany), butan-1-ol (Merck, Germany) and uranyl nitrate [UO_2_(NO_3_)_2_·6H_2_O; Aldrich, USA].

### Synthesis procedures

Two types of MSs were synthesized based on different silica sources including TEOS and Sodium metasilicate. 0.016 mole of TEOS, used as a precursor for silica, was added to the mixture of butan-1-ol and water, with the mole ratios of butan-1-ol/TEOS (10: 1) and H2O/TEOS (10: 1) under vigorous stirring at ambient temperature. When sodium metasilicate is used as a precursor for silica, firstly, a solution of sodium metasilicate and water should be prepared. After that, the obtained hydrogel was slowly dried at 100 °C for 3 h and then was refluxed in methanol over 24 h to complete dehydration. The resulting gel was then annealed at 600 °C for 4 h under atmospheric conditions^16,17^. As well as, two enhanced PFGs MSs were synthesized based on two mentioned silica sources. In this procedure, the phosphoric acid as a cost-effective source of PFGs was added to the synthesis process. In general, four MSs were synthesized which called TS (synthesized based on using TEOS as a precursor for silica), TS-Ph (synthesized based on using TEOS as a precursor for silica and the phosphoric acid as a source of PFGs), SMS (synthesized based on using sodium metasilicate as a precursor for silica), and SMS-Ph (synthesized based on using sodium metasilicate as a precursor for silica and the phosphoric acid as a source of PFGs)^15,18^.

### Characterization

Fourier transform infrared (FT-IR), X-ray diffraction (XRD), scanning electron microscopy (SEM), energy-dispersive X-ray spectroscopy (EDS), and Brunauer–Emmett–Teller (BET) analysis were used to characterize the synthesized MSs. XRD patterns were collected by XRD Bruker D8 Advance at 30 kV and 20 mA and Cu Kα radiation (λ = 0.1540598 nm). FT-IR spectra of synthesized samples were also recorded by TENSOR 27. The SEM (Zeiss-Sigma-VP-500 was used to study the morphology and particle sizes of synthesized MSs. The BET surface area and pore size distributions were obtained by BELSORP-mini analyzers (BEL Japan, Inc.). The element concentrations in adsorption experiments were performed by SPECTRO Genesis simultaneous charge-coupled device-based radially viewed inductively coupled plasma optical emission spectrometry (ICP-OES).

### Adsorption tests

Batch method was used to carry out the uranium and other elements sorption experiments. The adsorption tests were performed by preparing the different sample and standard solutions in various volumes and concentrations. The prepared AqS were contained of all the under studied elements with various pHs. Sodium hydroxide and diluted nitric acid were used to adjust the solution pHs of prepared samples.

The element concentrations were determined by ICP-OES before and after 5–60 min treatments. The element qe and its removal percentage (%) were estimated using the following equations, respectively:

The following equations were used to estimate the element qe and its removal percentage (%):

1 $${\rm{Adsorption}}\,{\rm{capacity}}\,(\mathrm{mg}/g)=\frac{({C}_{i}-{C}_{f})\ast V}{m}$$

2 $${\rm{Removal}}\,( \% )=\frac{({C}_{i}-{C}_{f})\ast 100}{{C}_{i}}$$

where *C*_*i*_ is the initial concentration and *C*_*f*_ is the concentration of the element at equilibrium after treatment with synthesized MSs. *m (mg)* and *V (ml)* are the mass of the adsorbent and the solution volume which was treated with MS, respectively^9,19^.

## Results and Discussion

**Figure 1:**
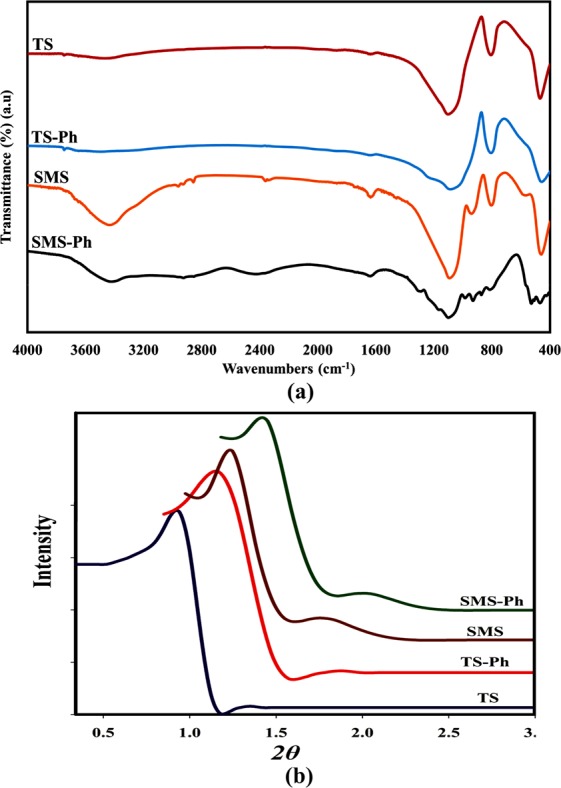
The (**a**) FT-IR spectra, (**b**) XRD patterns of the synthesized MSs.

The FT-IR spectra and XRD patterns of four synthesized MSs were shown in Fig. 1(a,b), respectively. The FT-IR spectra consisted of several main absorption bands which validated the synthesized MSs. The absorption bands of 410, 800, and 1060–1160 cm^−1^ are referred to one the main groups of IR absorption bands which are related to the ν_as_(Si–O–Si) frequencies. The P-containing groups were induced a new group of absorption band which are related to ν(P = O) frequencies. They usually concentrated around 1241 cm^−1^ on the FT-IR spectrum. These absorption bands are better observed in TS-Ph and SMS-Ph samples. Other main groups of observable absorption bands in the most IR spectra are related to the H_2_O related frequencies. The deformation vibrations of H_2_O are mostly located in the absorption bands around ‌ 1630 cm^−1^. A wide absorption band above 3000 cm^−1^ is usually related to the stretching vibrations of OH^20–22^. The XRD patterns of four synthesized MSs illustrated an intense diffraction peak in the range of 0.7°–0.9° which is related to (100), and one slight diffraction peak in the range of 1.2°–1.4° which is related to (110). The presence of these kinds of peaks indicated and confirmed the hexagonal structure (the p6m symmetry group) of synthesized MSs^13,23^.

**Figure 2:**
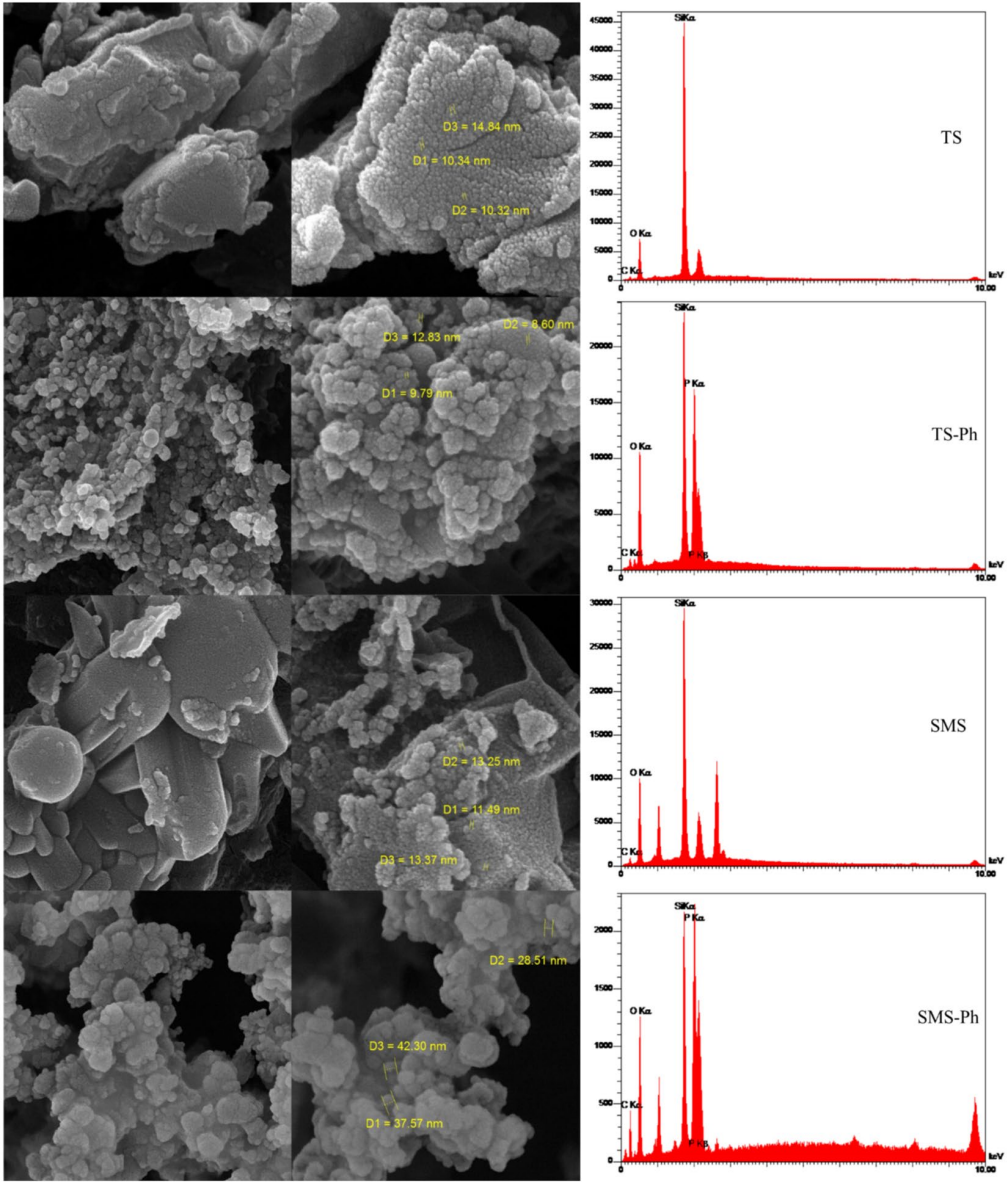
(**a**) SEM images and (**b**) EDS diagrams of the synthesized four synthesized MSs B, D, and B′.

SEM images and the EDS diagrams of four synthesized MSs of TS, TS-Ph, SMS, and SMS-Ph were shown in Fig. 2(a,b). From the SEM images, it can be deduced that the synthesized MSs have the nanostructure morphology with the regular and spherical shape. Figure 2(b) illustrates the EDS analysis of four synthesized MSs, exhibiting the presence of phosphorus in the synthesized MSs of TS-Ph, and SMS-Ph which can cause to form PFGs which were confirmed by FT-IR spectra. It is expected that the presence of PFGs will increase uranium qe. The uranium qe and its removal percentage by four synthesized MSs were summarized in Table 1. Adding phosphoric acid to the synthesis process considerably increased the uranium qe and removal percentage, which are reflective of the high efficient of the synthesized adsorbents. By comparing the adsorption results, the synthesized adsorbents based on sodium metasilicate (SMS and SMS-Ph) have adsorbed more uranium ions than the synthesized adsorbents based on TEOS (TS and TS-Ph), respectively. They also showed that sodium metasilicate based adsorbent (SMS-Ph) lead to creating better conditions to enhance PFGs compared to other silica-based adsorbents (TS-Ph).

**Table 1:** The uranium qe and its removal percentage by four synthesized MSs.

Sample	Adsorbent mass used (mg)	Volume of solution used (mL)	U qe (g-U/kg-ads)	% of U adsorbed
TS	5	50	45.5	9.3
TS-Ph	5	50	450.0	53.4
SMS	5	50	71.9	14.6
SMS-Ph	5	50	820.7	92.7

**Figure 3:**
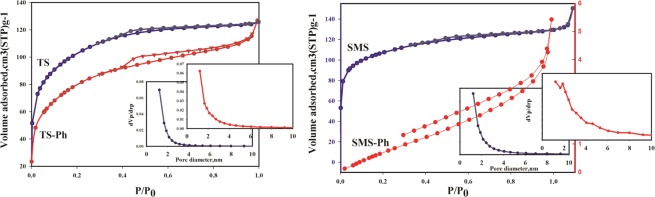
The N2 adsorption–desorption isotherms of the phosphoric acid based synthesized MSs including BET surface areas and PSDs.

Figure 3 shows the N_2_ sorption-desorption isotherm and pore size distribution (PSD) of four synthesized adsorbents which were measured at −196 °C by BELSORP mini analyzers. The synthesized samples were pretreated at 300 °C for 5 h. As shown in this Figure, the adsorption-desorption isotherms demonstrate type IV isotherms which are accompanied by type H4 hysteresis loop [37]. The PSD of the synthesized MSs is revealed in Figure inside Fig. 3, indicating that almost all the synthesized MSs has the PSD around 1.2 nm. The BET and Barrett–Joyner–Halenda (BJH) methods were utilized to compute the specific surface area (SSA) and PSD curve, respectively. The adsorption parameters of synthesized MSs including SSA, total pore volume and mean pore diameter were summarized in Table 2. As shown in this table, the SSA of SMS sample is more than the SSA of TS sample. It is also confirmed by the adsorption results which shows the higher qe of SMS with the higher SSA (Table 1). On the other hand, the results show that although SMS-Ph has the lower SSA compared to TS-Ph, it has the higher uranium qe. The enhanced functionalized of SMS samples by phosphoric acid (SMS-Ph) is the main reason of higher qe. Enhanced PFGs and created chelating on the synthesized MSs based on SMS shows the better surface modification which leads to the lower specific surface area. In most previous studies, SSA and pore size is a major factor for suitability of an absorbent, however, this statement didn’t confirm by obtained results. Therefore, it can be claimed that the enhanced functional groups are the main factor to achieve higher qe. This claim can be confirmed by the following investigations. JinHyeong Lee *et al*. studied the effects of increasing concentration of APTMS as a functional group agent on the surface area and adsorption capacity of mesoporous silica to remove Cr from aqueous solution. Their results shows that by increasing functional groups, the surface area was decreased and Cr adsorption capacity was increased [28]. Shengpan Peng *et al*. modified mesoporous silica nanoparticles by APTES functional group agent. Their results clarified functionalization with APTES leads to decrease surface area and increase adsorption capacity of target element [29].

**Table 2:** The adsorption and structural parameters of four synthesized adsobents.

sample	S_BET_,m^2^/g	Vs,cm^3^/g	dm, nm
TS	367.31	0.19	2.1
TS-Ph	283.98	0.19	2.73
SMS	384.64	0.23	2.41
SMS-Ph	4.28	0.008	7.73

Evidence indicates that the addition of phosphoric acid plays an essential role in functionalization of mesoporous silica, and consequently reducing the surface area. Khadijeh Beigom Ghoreishi *et al*. in a same research showed this reduction on the surface area by increasing phosphoric acid concentration [17]. The main factor contributed in enhancing uranium adsorption is O-P=O functionalization agents in different phosphonic functional groups agents in which phosphoric acid is the only source for providing O-P=O agents in this research.

**Figure 4:**
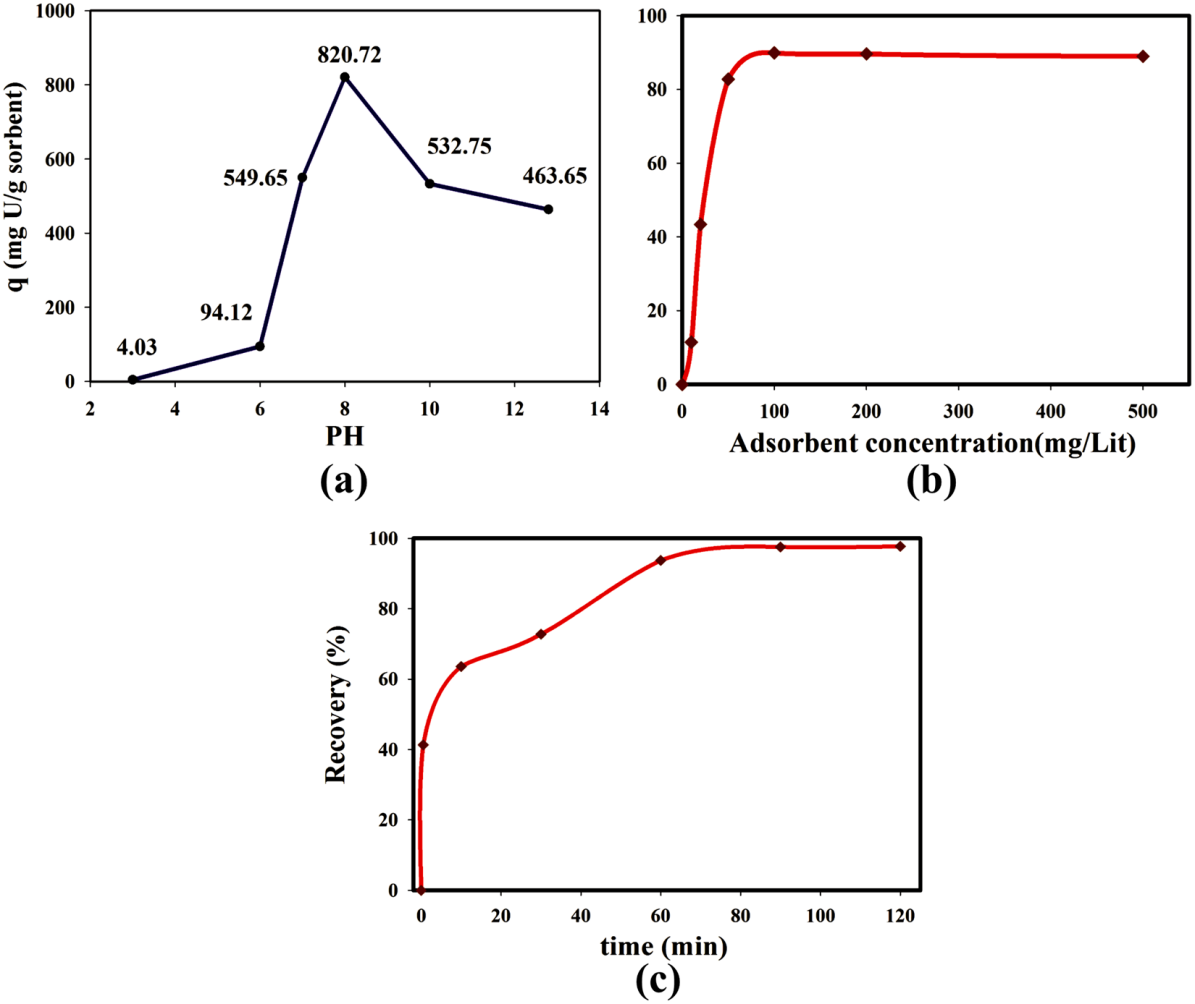
The effects of (**a**) the solution pH, (**b**) the adsorbent concentration on the uranium recovery percentages, (**c**) the contact time on the uranium recovery percentages for uranium adsorption by the synthesized sample of SMS-Ph.

Several different parameters including solution pH, adsorbent concentration and contact time influence on the uranium qe and percentages of uranium recovery in the uranium sorption experiments. Since that the maximum adsorption was achieved by SMS-Ph, this synthesized adsorbent was used to investigate the influences of mentioned effective parameters on the uranium qe and uranium recovery percentages. Figure 4 shows the obtained results of uranium qe and uranium recovery percentage variations by solution pH, the adsorbent concentration, and contact time, respectively. Figure 4(a) shows the uranium qe as a function of the solution pH, which indicated the maximum uranium qe of 820.7 mg/g was achieved at a solution pH of nearly 8. It also deduced that the synthesized adsorbent of SMS-Ph is the best alternative for uranium adsorption from the nearly neutral solutions such as seawater. The effect of adsorbent concentration on uranium recovery percentages is shown in Fig. 4(b). The adsorbent concentrations which were used in the sorption experiments was varied from 1 mg- 50 mg. The results indicated that the sorption reaches equilibrium at its maximum uranium recovery percentages of 90% at 60 min of contact time, room temperature and a solution pH of nearly 8. Figure 4(c) shows the influence of contact time of 0.5–90 min on uranium recovery percentages which indicated that the sorption reaches its equilibrium after 60 min. achieving around 40% of recovery percentages at the 30 seconds means the synthesized MSs exhibits a considerable potential adsorbent for using in the sorption column.

**Figure 5:**
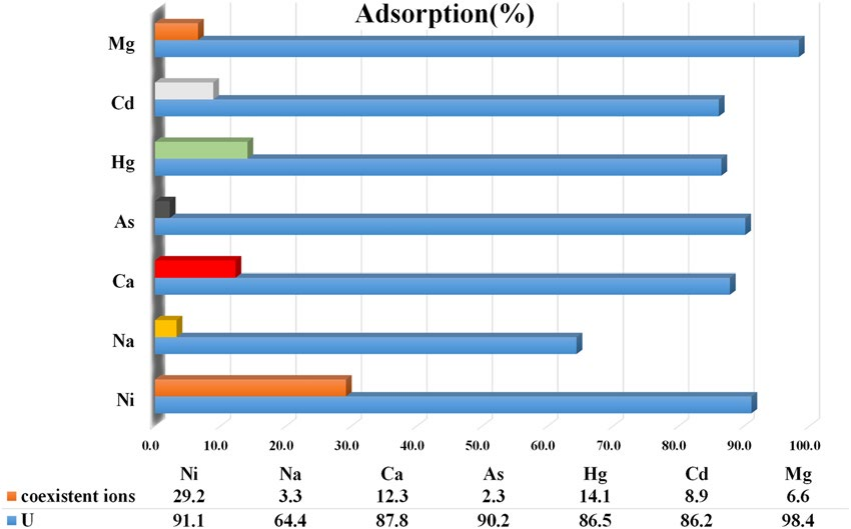
The uranium recovery percentages of the synthesized adsorbent of SMS-Ph in the presence of other elements including Mg, Cd, Hg, As, Ca, Na and Ni.

Synthesis of a selective uranium adsorbent was another significant achievement in this research. The uranium recovery percentages of the synthesized adsorbent of SMS-Ph in the presence of other elements including Mg, Cd, Hg, As, Ca, Na and Ni are shown in Fig. 5. The sorption experiments were performed by using 5 mg adsorbent concentration at 60 min of contact time, room temperature and a solution pH of around 8. The results indicated that the MS (SMS-Ph) exhibits the acceptable selectivity for uranium adsorption in the presence of mentioned elements which conform to achieve to the second aim in this work. The selective property of the synthesized MS was also examined in the presence of a high concentration of Cl^−^ ions by dissolving NaCl in water to simulate its behavior in the salty environment such as sea water. The result shows the uranium recovery of 92.6% in the presence Cl^−^ ions which also confirm the synthesis of the best alternative for uranium adsorption from the nearly neutral solutions such as seawater.

Synthesis of an economic adsorbent was another and the main purpose of this and approximately all researches which is essential criteria to define an appropriate adsorbent. The economic possibility of the adsorbent fabrication is defined as the cost per unit of mass of the raw materials which were used in the synthesis process. Several activities and factors were used to reduce the costs in the synthesis process of the enhanced functionalized SMS-Ph adsorbent which were (1) removing the silane agents, (2) using sodium metasilicate as a low-cost source of silica, (3) using the Sol-gel method as an unexpansive synthesis technique. Since in the Sol-gel method, no need to use the template polymer, the synthesis process duration decreases efficiently. Moreover, removing template polymer which is one of the time-consuming processes was eliminated. However, using template method and template polymer cause to increase the costs in the synthesis process. Table 3 summarized the details of the costs in the synthesis processes of two different MS adsorbents based on the Sol-gel method and template method (our previous work) to confirm the synthesis of a cost-effective adsorbent. The price presented based on the cost of the amount of the materials which were used in one synthesis experiment, i.e. the estimated price for the amount of the used sodium metasilicate in the sol-gel method which was 2 gram was 0.1122 EUR (1 kg of sodium metasilicate was purchased 56.1 EUR). The estimated total price indicated the used method considerably decrease the synthesis cost around quarter. Needless to mention, this method didn’t have the complexity of template method and effectively reduces synthesis time and processes^16^.

**Table 3:** The details of the costs in the synthesis processes of two different MS adsorbents based on the Sol-gel method and template method.

Sol-gel method (Materials)	Price (EUR) based on the amount of the used materials	Template method (Materials)	Price (EUR) based on the amount of the used materials
Sodium metasilicate	0.1122	sodium metasilicate	0.1276836
Ortho-phosphoric acid	0.05252	DPTS	0.6932
butan-1-ol	0.19575	Pluronic P123	0.2288
		acetic acid	0.28704
Total price (for 6 gr synthesized adsorbent)	0.36047		1.3367236
Total price (for 1 kg synthesized adsorbent)	60.078		222.787

**Figure 6:**
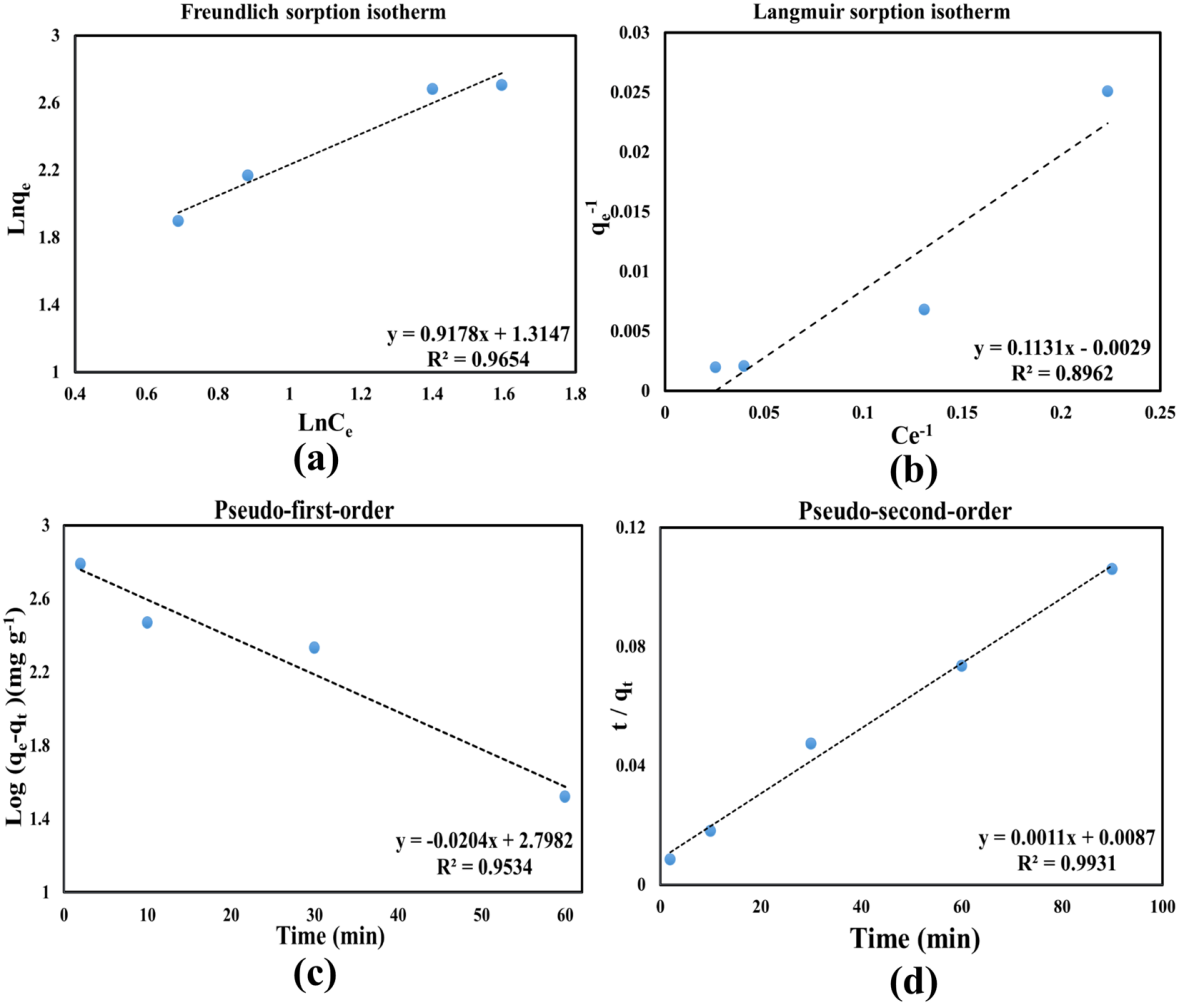
(**a**) The Langmuir and (**b**) Freundlich adsorption isotherms, (**c**) The pseudo-first- and (**d**) second order kinetic models for uranium adsorption on synthesized MS sample of S′.

Understanding the sorption mechanism and studying the related sorption isotherms are the fundamental issues in synthesis and survey of an adsorbent. In this regards, the Langmuir and Freundlich models are usually used to analyze the adsorption results in the equilibrium condition. Figure 6(a,b) show the Langmuir and Freundlich sorption models for uranium adsorption on the synthesized adsorbent of SMS-Ph at optimum pH of approximately 8, respectively. The formation of a homogeneous monolayer of considered element ion on the outer surface of the adsorbent is the basic concept of the Langmuir isotherm. The Langmuir model is expressed

3 $$\frac{{C}_{e}}{{q}_{e}}=\frac{{C}_{e}}{{q}_{m}}+\frac{1}{{q}_{m}{K}_{l}}$$

where *q*_*m*_ is the maximum qe of the monolayer of the sorbent (*mg/g*) at equilibrium, and *K*_*L*_ is the Langmuir adsorption constant (*L/mg*). The Freundlich isotherm is usually expressed as the model for heterogeneous adsorption as below:

4 $$\mathrm{ln}\,{{q}}_{e}=\,\mathrm{ln}\,{{k}}_{F}+\frac{1}{n}ln{C}_{e}$$

where *k*_*F*_ is the Freundlich isotherm constant and n is the dimensionless heterogeneity factor. Table 4 summarizes the resulted parameters and the correlation coefficient of the Langmuir and Freundlich sorption models. The results indicated the approximately 344.83 mg/g adsorption capacity with the low correlation coefficient of 0.896 for the Langmuir adsorption isotherm. The important term in the Freundlich model is the inverse of heterogeneity factor (*1/n*) and estimated as the slope of the Freundlich model curve. The higher correlation coefficient of 0.9654 and the near unity of the inverse of heterogeneity factor indicated that the Freundlich isotherm is the favorable isotherm for uranium adsorption by synthesized adsorbent of SMS-Ph. The variation of the inverse of heterogeneity factor is between 0 and 1 which is associated with the sorption process. The near unity of the inverse of heterogeneity factor indicated the adsorbent us consistent with a cooperative adsorption^24,25^. As well as, the pseudo-first- and second-order kinetic models were used to confirm measured sorption capacity and interpret the sorption kinetics of uranium on the synthesized adsorbent of SMS-Ph. Figure 6(c,d) show the pseudo-first- and second-order kinetic models for uranium adsorption on the synthesized adsorbent of SMS-Ph at optimum pH of approximately 8, respectively. The Eqs (5) and (6) are explained the pseudo-first- and second-order kinetic models, respectively:

5 $$ln({q}_{e}-{q}_{t})=\,\mathrm{ln}\,{{q}}_{e}-{k}_{1}t$$

6 $$\frac{t}{{q}_{t}}=\frac{t}{{q}_{e}}+\frac{1}{{q}_{e}^{2}{K}_{2}}$$

where *q*_*e*_ (*mg g*^*−1*^) and *q*_*t*_ (*mg g*^*−1*^) are the amounts of uranium adsorbed in synthesized MS sample (*mg g*^*−1*^) at equilibrium and at time of *t* (*min*), respectively, and k_1_ (*min*^*−1*^) and k_2_ (*g mg*^*−1*^ *min*^*−1*^) are the sorption rate constant of first and second-order kinetic model, respectively. The results indicated that the correlation coefficient of the pseudo-second-order model was obtained 0.993 which shows the accurately matches with the experimental kinetics data. The uranium qe of 909.091 *mg/g* was estimated by this model at the equilibrium condition which shows the highest qe by the synthesized adsorbent of SMS-Ph. Table 5 summarized the calculated parameters of pseudo-first- and second-order kinetic models^26,27^.

**Table 4:** The resulted parameters and the correlation coefficient of the Langmuir and Freundlich isotherms.

LANGMUIR isotherm	q_m_ (mg/g)	k_L_ (L/mg)	R^2^
	344.83	0.0256	0.8962
**FREUNDLICH isotherm**	**n**	**k**_**f**_	**R** ^**2**^
	1.089	20.6395	0.9654

**Table 5:** The extracted parameters of pseudo-first- and second order kinetic models.

Kinetics model	Pseudo-first-order			Pseudo-second-order		
	q_e_ (mg/g)	k_1_ (min^−1^)	R²	q_e_ (mg/g)	k_2_ (g/mg min)	R²
	16.426	0.0204	0.953	909.091	0.00013908	0.993

## Conclusion

Two kinds of MS adsorbents based on the different silicon sources including TEOS and sodium metasilicate were synthesized by Sol-Gel method and modified by phosphoric acid to enhance PFGs. The synthesis of these adsorbents was accomplished with the approach to achieving optimum performance characteristics including higher adsorption efficiency, selectivity, and materials availability, and low- cost production. Different analysis methods including FT-IR, XRD, SEM, EDS, and BET confirmed the characterization of the synthesized adsorbents. The sorption results showed that SMS-Ph which was synthesized based on sodium metasilicate and modified by phosphoric acid exhibited the highest uranium qe of 820.7 mg/g at a solution pH of approximately 8 which indicated the synthesized adsorbent is the best alternative for uranium adsorption from the nearly neutral solutions such as seawater. The results indicated that despite most previous studies which introduced the SSA and pore size as a major factor for suitability of an absorbent, enhanced PFGs and created chelating on the synthesized MSs are the main factor to achieve higher qe. SMS-Ph also showed the acceptable selectivity for uranium adsorption from the AqS such as industrial wastewater. Reduction the synthesis costs from 222.787 EUR/kg for the template method to 60.078 EUR/kg for Sol-gel method have also confirmed the synthesis of a cost-effective adsorbent.
